# Resource‐Related Interactions Between Wolverines and Brown Bears in Scandinavia

**DOI:** 10.1002/ece3.74553

**Published:** 2026-10-30

**Authors:** Rick W. Heeres, Jens Persson, Johanna Heeres, Andrés Ordiz, Jonas Kindberg, Malin Aronsson

**Affiliations:** ^1^ Grimsö Wildlife Research Station, Department of Ecology Swedish University of Agricultural Sciences Riddarhyttan Sweden; ^2^ Department of Natural Sciences and Environmental Health University of South‐Eastern Norway Bø Norway; ^3^ Wildlife Institute Forest Research Institute of Baden‐Württemberg Freiburg im Breisgau Germany; ^4^ Area of Zoology, Department of Biodiversity and Environmental Management, Faculty of Biology and Environmental Sciences University of Léon Léon Spain; ^5^ Norwegian Institute for Nature Research Trondheim Norway; ^6^ Department of Wildlife, Fish, and Environmental Studies Swedish University of Agricultural Sciences Umeå Sweden

**Keywords:** *Gulo gulo*, large carnivore guild, resource competition, Scandinavia, scavenging, *Ursus arctos*

## Abstract

Ongoing recovery of large carnivore species into their former ranges increases the occurrence of interactions among them. The recovering Scandinavian wolverine ( *Gulo gulo* ) population has recently expanded southwards and is increasingly overlapping with the core of the brown bear ( *Ursus arctos* ) range. Both species are facultative scavengers that feed on moose ( *Alces alces* ) carcasses, which are an important resource all year round for wolverines and seasonally for bears. Reliance on the same resource may lead to interactions. We monitored five GPS‐collared wolverines in central Sweden to investigate feeding sites with food remains and determine bear activity. In addition, we used motion‐triggered cameras to monitor wolverine and bear activity at three wolverine rendezvous sites (i.e., temporary sites where females keep cubs) and at a recently bear‐killed adult moose carcass. We found 115 wolverine feeding sites, of which 25 (22%) showed signs of bear activity. At 24 sites we found remains from adult moose carcasses, five presumably bear‐killed of which two were fresh (< 1 week). Based on GPS‐data, wolverines repeatedly used these recently bear‐killed moose for periods ranging from a few hours to multiple days. Bear activity was documented at two of the three presumed rendezvous sites used by a female wolverine with cubs. Camera data also documented repeated visits by at least three different wolverines at the presumed bear‐killed moose carcass, including visits short before and after bear activity at the site. Our results illustrate that wolverines and bears overlap and share food resources, potentially leading to direct and/or indirect interactions. These observations suggest that co‐occurrence between these two species create both feeding opportunities and potential risks for wolverines, which deserves further research.

## Graphical abstract

Some areas are witnessing the return of several large carnivore species to their former ranges. In this context, northern Eurasia in general and Scandinavia in particular have become a hotspot for large carnivore recovery; as wolverines, brown bears, wolves, and lynx are increasingly sympatric. In this study, we provide empirical documentation of interactions between wolverines and bears based on both GPS‐radiotracking and motion‐triggered camera trap data.

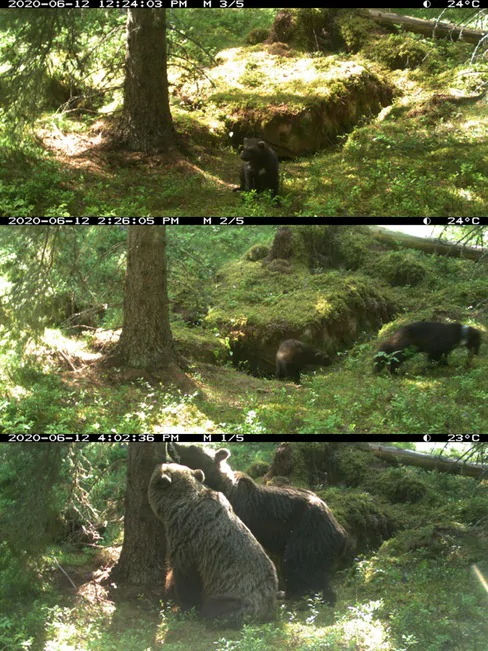

## 1 Introduction

The return of large carnivores can lead to major changes in ecosystems (Estes et al. 2011) and increasing overlap between carnivore species may lead to the renascence of interspecific interactions. Competition for resources, via interference competition, exploitative competition, and/or kleptoparasitism, and altered habitat use have been documented in different carnivore guilds (Davis et al. 2018; Milleret et al. 2018; Ordiz et al. 2015; Tallian et al. 2017; Vanak et al. 2013). Species can also facilitate each other by generating scavenging opportunities (Binder et al. 2026; Krofel et al. 2012; Mattisson, Andrén, et al. 2011; Ordiz et al. 2020; Tallian et al. 2017), which in turn can increase the risk of direct encounters and thus aggressive interactions (Binder et al. 2026). It is therefore important to understand the mechanisms that allow coexistence within the same guild (Dröge et al. 2017), and the potential impact of interspecific interactions at the ecosystem level (Haswell et al. 2016).

Large carnivore populations have been recolonizing their former ranges in Europe during the last decades (Chapron et al. 2014), and in Scandinavia four large carnivores (wolf, *Canis lupus* ; brown bear, *Ursus arctos* (hereafter: bear); Eurasian lynx, *Lynx lynx* ; and wolverine, *Gulo gulo* ), have been expanding their distribution (Chapron et al. 2014; Persson et al. 2023). Especially due to the continued expansion of wolverines in Scandinavia (Persson et al. 2023), the probability of co‐occurrence and interactions with the other large carnivores are increasing. Due to intense human persecution in the 1900s, the wolverine population was diminished and confined to northern alpine areas (Flagstad et al. 2004). Following population recovery, wolverines are now recolonizing the boreal forest towards the east and south (Aronsson and Persson 2017; Persson et al. 2023). Due to the range expansion, the southern part of the wolverine population increasingly overlaps with the distribution of bears and wolves (Bischof et al. 2020).

The wolverine is a facultative predator and scavenger (Mattisson et al. 2016). They benefit from lynx in the Sámi reindeer husbandry area in the north of Sweden (Mattisson, Andrén, et al. 2011; Mattisson, Persson, et al. 2011) and wolves may have facilitated the wolverine expansion further south as both carnivores provide scavenging opportunities (Nordli et al. 2024; van Dijk, Andersen, et al. 2008; van Dijk, Gustavsen, et al. 2008). At the same time, larger and pack‐living wolves also pose a risk for wolverines due to intraguild aggression (Wallace et al. 2021; Young et al. 2023). However, little is known about interactions between wolverines and bears in Scandinavia.

Bears are also opportunistic scavengers and predators, and prey on ungulate calves as an important food source in spring and early summer (mainly moose, *Alces alces* , but also reindeer, *Rangifer tarandus* in northern Sweden; Ordiz et al. 2020; Sivertsen et al. 2016). Additionally, bears sometimes prey on yearling or adult moose, mainly in April and May (Dahle et al. 2013). In summer, bears focus on foraging ants and vegetation, and later in summer berries (Ordiz et al. 2020). While bears can provide scavenging opportunities for wolverines, they might also compete for the same scavenging resources. Additionally, wolverines regularly cache parts of killed prey or carrion as an adaptation to unpredictable food availability (van der Veen et al. 2020), and these might be kleptoparasitized by bears. Therefore, it is likely that wolverine‐bear interactions may predominantly occur around food sources, such as carcasses or cached food. Furthermore, similar to wolves, bears may pose a threat to wolverines (Wildlife Conservation Society 2003).

In this study, we used fine‐scale monitoring of GPS‐collared wolverines, subsequent field investigations, and camera traps to provide empirical documentation of interactions between wolverines and bears in central Sweden. We therefore provide further insights into large carnivore co‐occurrence in boreal latitudes.

## 2 Material and Methods

### 2.1 Study Area

Our study area was in central Sweden (Figure 1), including the counties of Jämtland, Västernorrland, and Gävleborg. Most of the study area is covered by intensively managed boreal forest, consisting mainly of Norway spruce ( *Picea abies* ) and Scots pine ( *Pinus sylvestris* ), interspersed with deciduous species such as birch (*Betula* sp.), and altitude ranges from 100 to 830 m. Due to forestry activities the landscape is interspersed with gravel roads (1 ± 0.5 km/km^2^), and logging is the major commercial activity (Ordiz et al. 2014). In general, the human density is low, with about 1–7 inhabitants/km^2^. The wolverine density in this part of south‐central Sweden is relatively high (Bischof et al. 2020), and the bear density is ±60 bears/1000 km^2^ (Bischof et al. 2020). Moose are the most abundant ungulate (0.7–1.6/km^2^; Sand et al. 2016), but roe ( *Capreolus capreolus* ) and red deer ( *Cervus elaphus* ) also occur at very low densities. Wolves and lynx occur at low densities in the area (Andrén et al. 2020; Bischof et al. 2020).

**FIGURE 1:**
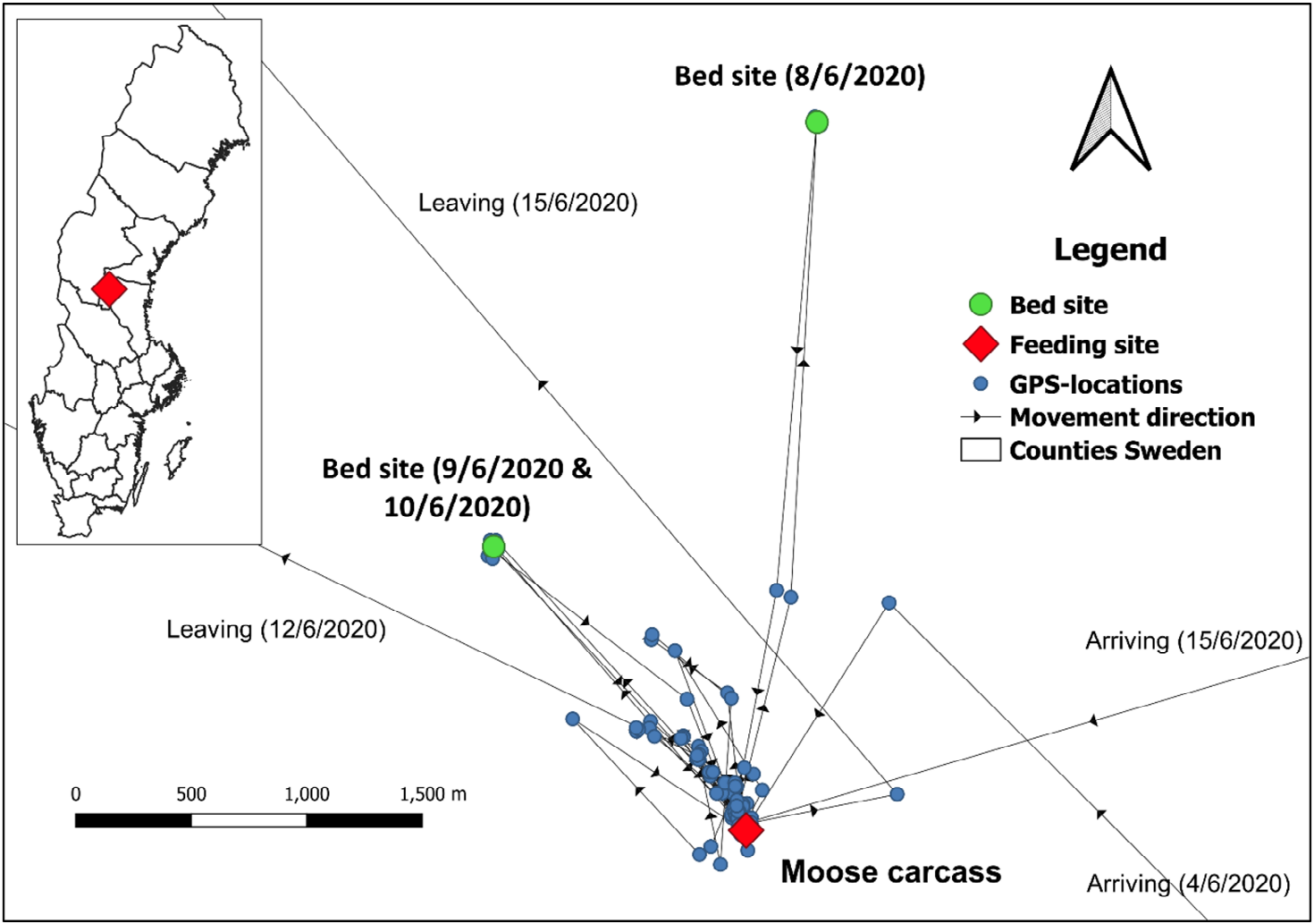
Wolverine activity based on GPS‐location data (blue dots and black lines) surrounding a recently (< 1 week) bear‐killed moose in central Sweden (top left corner). The wolverine spent time at the feeding site (red square) during two periods: The first one lasted about 7 days (from 4/6/2020 until 12/6/2020) and it used several bed sites (green dots) within < 3 km from the carcass; the second period lasted approximately 2 h (15/6/2020). Numbers in the figure refer to the dates arriving, leaving, and the use of bed sites. Figure S1 shows another example of wolverine activity surrounding a recently killed moose carcass.

### 2.2 Wolverine Captures and Monitoring

Wolverines were captured by using log‐box traps, immobilized with a mixture of ketamine and medetomidine and equipped with GPS‐collars (Vertex lite GSM‐collars, Vectronic Aerospace GmbH, Berlin, Germany). All captures and handling of wolverines were done following pre‐established protocols (Arnemo and Evans 2017) approved by the Animal Ethics Committee for northern Sweden and fulfilling the ethical requirements for research on wild animals (protocol number A40‐2018).

### 2.3 GPS‐Cluster Investigations to Find Wolverine Feeding Sites

We monitored five wolverines equipped with GPS‐collars programmed to take one location every hour during two study periods of 3 weeks (May 28–June 17 in 2019, and May 22–June 11 in 2020; due to collar malfunctioning one wolverine was monitored for 9 days; Table 1, Table S1). Clusters of GPS‐positions were defined as ≥ two positions within 200 m, identified using a script designed for standardized GPS cluster analysis which is now available as the *ClusterApp* (Heeres et al. 2024) in R (R Development Core Team 2024). GPS‐clusters were investigated in the field within 3 days after identification, which was a trade‐off between detecting signs of wolverine activity while minimizing potential disturbance of the study animals. The method to investigate clusters in the field for predation studies is an established method used for several carnivore species in Scandinavia (cf. Mattisson, Andrén, et al. 2011; Mattisson et al. 2016; Ordiz et al. 2020; Rauset et al. 2012; Støen et al. 2022).

**TABLE 1:** Overview of sites that were classified as feeding sites, as well as the number of sites with confirmed bear activity (both in numbers and percentages), per wolverine (*n* = 5) during the study period in 2019–2020 in central Sweden.

Wolverine	Sex	Monitoring days	# Sites	# Sites with bear	% Sites with bear
J19026	M	21	23	6	26
J19027	F	21	21	5	24
J16014	F	22	38	5	13
J20030	M	22	18	3	17
J20032	F	9	15	6	40
Total			115	25	22

All clusters were searched for food remains (i.e., whole carcass or remains such as bones or feathers; hereafter feeding site) or other signs of wolverine activity (i.e., if signs of a bed on the ground and/or wolverine hair was found, the site was registered as a potential bed site). One cluster could contain several feeding and bed sites that were documented separately. For all carcasses, we determined the potential cause‐of‐death based on signs at the site, for example, species‐specific marks such as tracks, hair, claw marks (carnivore killed) or sawn‐off bones (human caused death from hunting). For food remains, we determined the species based on remains of hair, bones, hooves, and feathers (Mattisson et al. 2016). One feeding site could include remains from several species. Furthermore, as wolverines frequently cache food remains, we registered feeding sites as potential cache site, if one or more body parts were hidden under boulders/logs or dug into a hole (van der Veen et al. 2020). At each site we searched for signs of bear activity, including scat, hair, and claw marks, as well as bear‐specific behavior such as covering a carcass with vegetation (Rauset et al. 2012).

### 2.4 Motion‐Triggered Cameras

During 2020, we selected four of the documented feeding sites to place motion‐triggered cameras (HyperFire PC800 professional; Reconyx Inc., Holmen, WI, USA) to document wolverine and bear activity. The sites were selected *ad hoc* during fieldwork; we were especially interested in the use of potential rendezvous sites and recent adult ungulate carcasses by wolverines. Three of the feeding sites were also suspected to function as rendezvous sites for one of the two GPS‐collared wolverine females (2 sites for *J20032* and 1 for *J16014*) known to have cubs at the time, as we found cached food remains, multiple potential bed sites, and cavities with wolverine hair inside (Table S2). Sites 1 and 2 were used by a female (*J20032*) with two cubs and Site 3 was used by a female (*J16014*) with one cub (Tables S1 and S2). Based on the photos, all sites were visited by the wolverine females and cubs after placing the cameras. During this time of the year and especially towards the end of the denning season, females use rendezvous sites where they keep their cubs for short periods of time (Magoun and Copeland 1998). Rendezvous sites are typically characterized by resting sites, protective structures such as boulders or logs, and often latrines and food remains. At the three rendezvous sites, no bear activity was found prior to the placing of the cameras. The fourth camera site was an adult moose carcass, presumably killed by a bear based on signs such as removed vegetation and trampling of the ground.

We set up one motion‐triggered camera per site and kept them at the locations until the end of the study period. Therefore, the deployment time varied among camera sites depending on when the site was found during the study period (Table S2). The cameras were placed at knee‐height and set to take a series of three photos when motion‐triggered (rapid‐fire setting), to be able to determine when individuals entered and exited the site. Each photo was reviewed manually to assess the presence of the focal species and to determine the number of individuals. Consecutive photos within 15 min were defined as one visit. Visit duration was calculated using the first and last photo of a visit. The time between visits of wolverines and bears was calculated using the last photo of a wolverine visit and the first photo of a bear visit at the same site, and vice versa. GPS‐collared wolverines were individually identifiable by their collars, whereas our camera setup did not allow for individual identification of uncollared wolverines based on unique chest patterns. For GPS‐collared females we knew the number of offspring, and cubs were identified due to their smaller size.

## 3 Results

### 3.1 Wolverine Feeding Sites

During the study periods, we investigated 165 clusters of wolverine GPS positions (Table S1) and found a total of 161 sites with signs of wolverine activity at 91 of the clusters; 115 feeding sites (108 and 7 with remains from one or more prey species, respectively), and 46 bed sites (Table 1). At the remaining 74 clusters, we did not find any wolverine activity. Of the 115 feeding sites, 22 were identified as potential cache sites.

Moose remains were the most frequent food remains found at feeding sites (*n* = 80), with remains from adult moose, moose calves, and moose of unknown age at 24, 35, and 21 sites, respectively. The remaining feeding sites contained remains from forest grouse ( *Lyrurus tetrix* or *Tetrao urogallus* ; *n* = 30), lagomorphs (*n* = 1), other bird species (*n* = 1), unknown ungulates (*n* = 5), and livestock (*n* = 6; found at sites where normally only hunting remains are discarded). As feeding sites could contain remains from multiple prey species, the number of food remains found (*n* = 123) is larger than the total number of feeding sites (*n* = 115). Most feeding sites only contained scattered remains; hence we could not assess the total number of adult moose and calves, and the number of feeding sites likely overestimates the number of individual moose, as remains from the same individual may have occurred at multiple feeding sites. Of the 16 clusters (24 feeding sites) with adult moose remains, 5 were likely killed by bears (of which 2 were recently killed carcasses < 1 week, for example, sign of struggle was visible in the vegetation), 3 originated from hunting remains (sawn‐off bones), and 8 had unknown death causes. It was not possible to indicate the cause of death for the sites with moose calf remains.

Bear activity was confirmed at 25 out of the 115 wolverine feeding sites (22%; Table 1) with remains from one (*n* = 23) or more prey species (*n* = 2). The 25 feeding sites included 7 with adult moose remains, 13 had moose calf remains, 2 with remains from moose of unknown age, 1 with remains from an unknown ungulate of unknown age, 3 with domestic cow remains, and 1 with black grouse remains. Of the 7 sites with adult moose remains, 5 were presumably killed by bear (including the 2 recently killed moose) and 2 originated from hunting remains. Based on the GPS‐locations, wolverines made 5 visits to the vicinity (< 3 km) of the two recently killed adult moose (Figure 1, Figure S1), with visits lasting from only a few hours up to multiple days (range = 2–173 h). The time between visits was 43 h on average (*n* = 3; range = 27–72 h). At one of the moose carcasses (Figure 1), we also placed a motion‐triggered camera (Figure 5).

### 3.2 Camera Sites Showing Wolverine and Bear Visits at Rendezvous Sites and Moose Carcass

We obtained a total of 15,112 photos (Table S2) from the 4 deployed motion‐triggered cameras during the study period (2049 of wolverines and 1999 of bears). The wolverine family groups were photographed at all 3 rendezvous sites (Sites 1–3: Table S2; Figures 2, 3, 4), during a total of 11 visits (5, 3, and 3 visits at Sites 1, 2 and 3, respectively). These included visits by either the female alone (6 visits, all lasting < 3 min) or the female together with cub(s) (5 visits; lasting on average 15 min (range = 2–42 min)). At Site 2 (Figure 3), one of the two cubs spent 16 min in front of the camera when they were left alone, after which it entered a cavity at the site and emerged again when the female returned. Consequently, the time that the female left the cubs alone at the rendezvous site was estimated to be about 2 h. Most visits to the sites by the female alone were at night, while most visits by the female together with cubs were during the day. At all sites the female either ate (Figures 2 and 4) or carried away the cached food (Figure 3).

**FIGURE 2:**
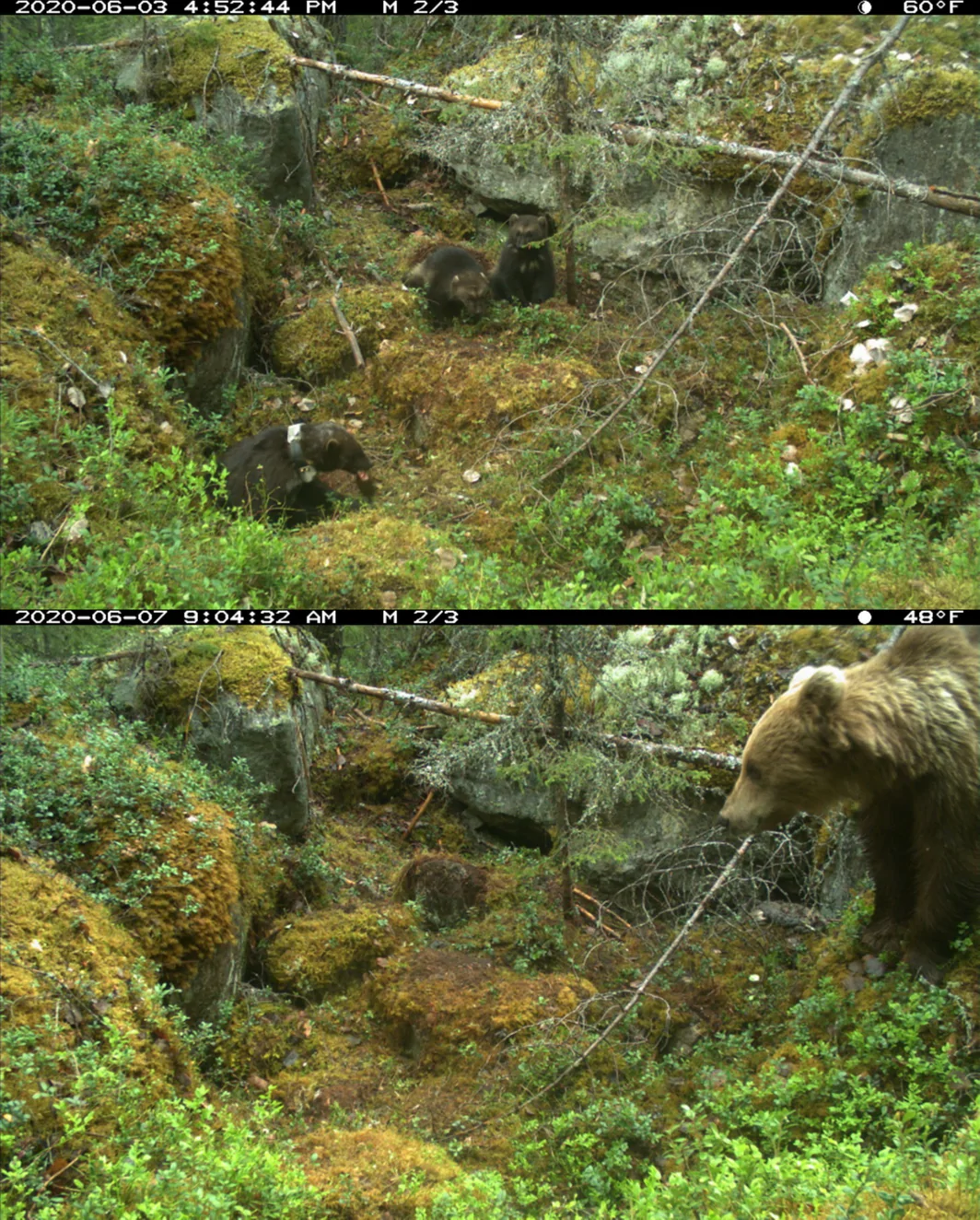
Photos from Site 1 (rendezvous site). The upper photo shows the female and cubs, where the female is eating a cached moose calf leg. The moose calf leg was found cached under the rocks when the site was investigated prior to setting the camera. During this visit (42 min), the cubs played together or sat next to the female. The lower photo shows a brown bear visiting the site (1 min), 4 days after the wolverine female and cubs.

**FIGURE 3:**
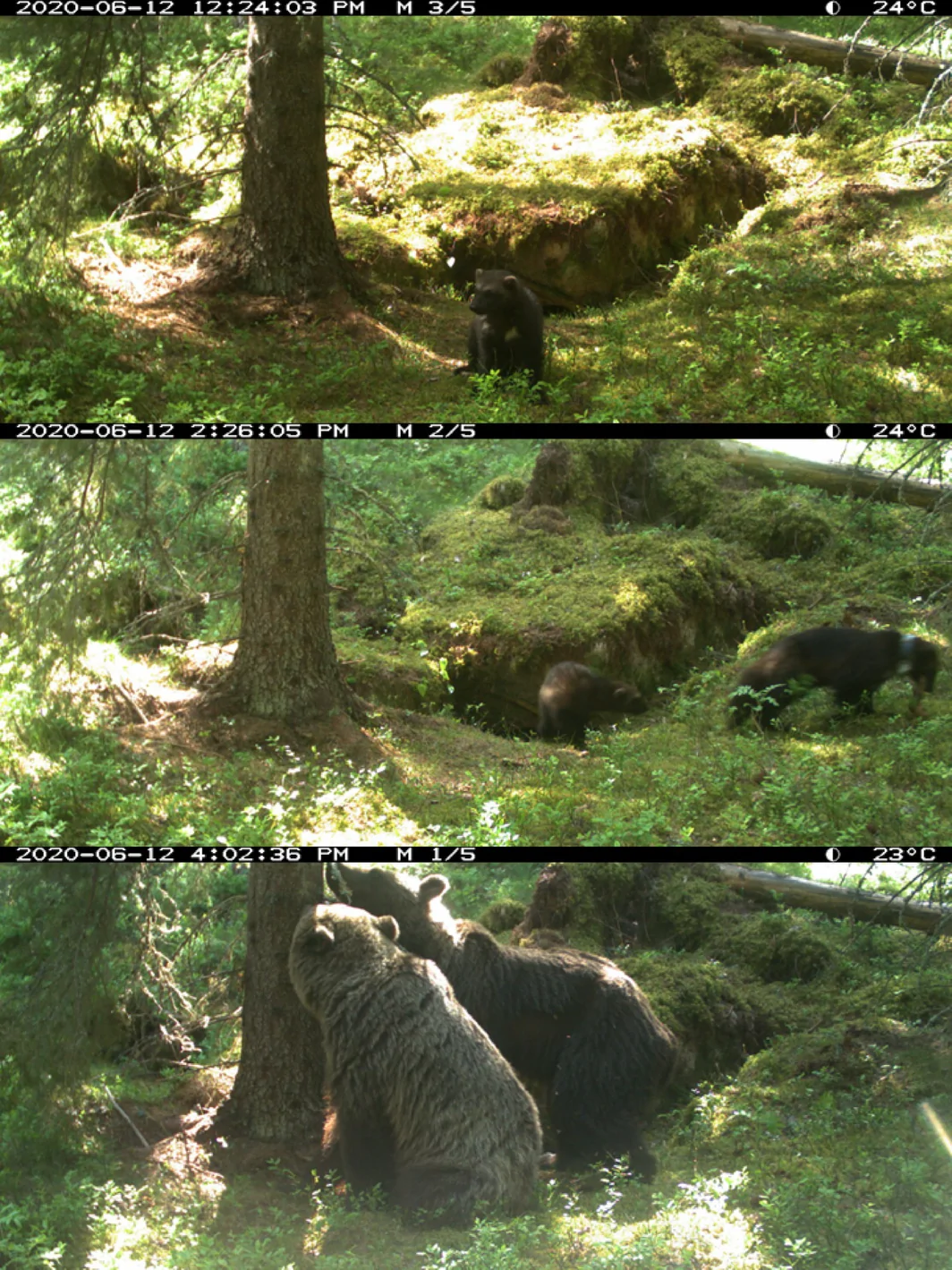
Photos from Site 2 (rendezvous site). The upper photo shows one of the cubs alone at the site. The female left the cubs alone at the site for about 2 h. One of the cubs was observed outside the cavity under the rocks for about 16 min during this period; investigating the area around the entrance. The cubs were never observed together. The middle photo shows when the wolverine female is leaving the site together with the cub/‐s after she was away for about 2 h, while carrying a cached moose calf leg. The moose calf leg was found cached when the site was investigated prior to setting the camera. The lower photo shows two brown bears visiting the site (5 min), approximately 1.5 h after the wolverine family left.

**FIGURE 4:**
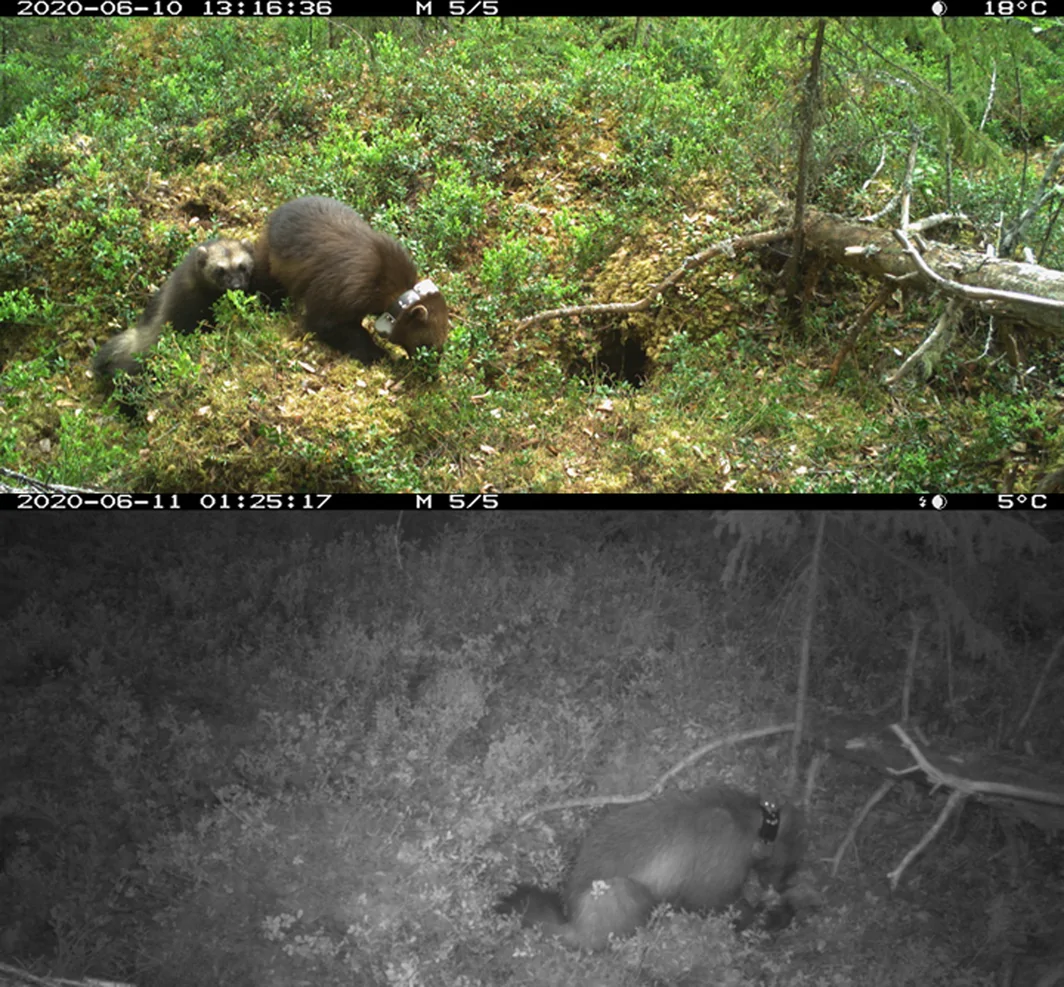
Photos from Site 3 (rendezvous site). The upper photo shows the wolverine female with a cub during the only visit together at the site (lasting about 2 min). The lower photo shows the female wolverine eating a cached moose calf leg. After feeding for about 3 min, the female left with the leg. The moose calf leg was found cached when the site was investigated in the field prior to setting the camera.

Bears were photographed at two of the rendezvous sites during 5 visits (Sites 1 and 2), with two bears together at one of the visits (Figure 3). The average duration of the bear visits was 3 min (range = 1–5 min). Bears were observed to investigate the sites, but we could not verify whether they scavenged any of the cached food. On average, bears visited the rendezvous sites 33 h (range = 1.5–84 h) after a wolverine visit, and a wolverine visited the sites 89 h after a bear visit (range = 3–234 h).

At the adult moose carcass (Site 4: Table S2, Figure 5), we documented at least three different wolverines during 18 visits (average duration 18 min, range = 1–44 min): 6 visits by the GPS‐collared male, which was together with one uncollared individual during 4 of these visits, and 12 visits by uncollared wolverines. The uncollared wolverines included one larger and one smaller individual that were together during 3 of the visits, presumably a mother with a cub. The camera setup did not allow for individual identification of the uncollared wolverines, but their joint visits and size difference confirmed the presence of at least two different uncollared individuals. The wolverines were observed feeding during 6 of the visits, leaving with carcass pieces during 2 visits, interacting during 4 visits, and either moving through or checking the area during 7 visits. We did not observe any aggressive behavior between the collared male and the uncollared individual during the 4 joint visits, nor between the two uncollared individuals. The time between visits for the collared male was about 5.5 h (*n* = 6, range = 1–13.5 h). We were unable to determine the time between visits for the uncollared individuals due to potential misidentification.

**FIGURE 5:**
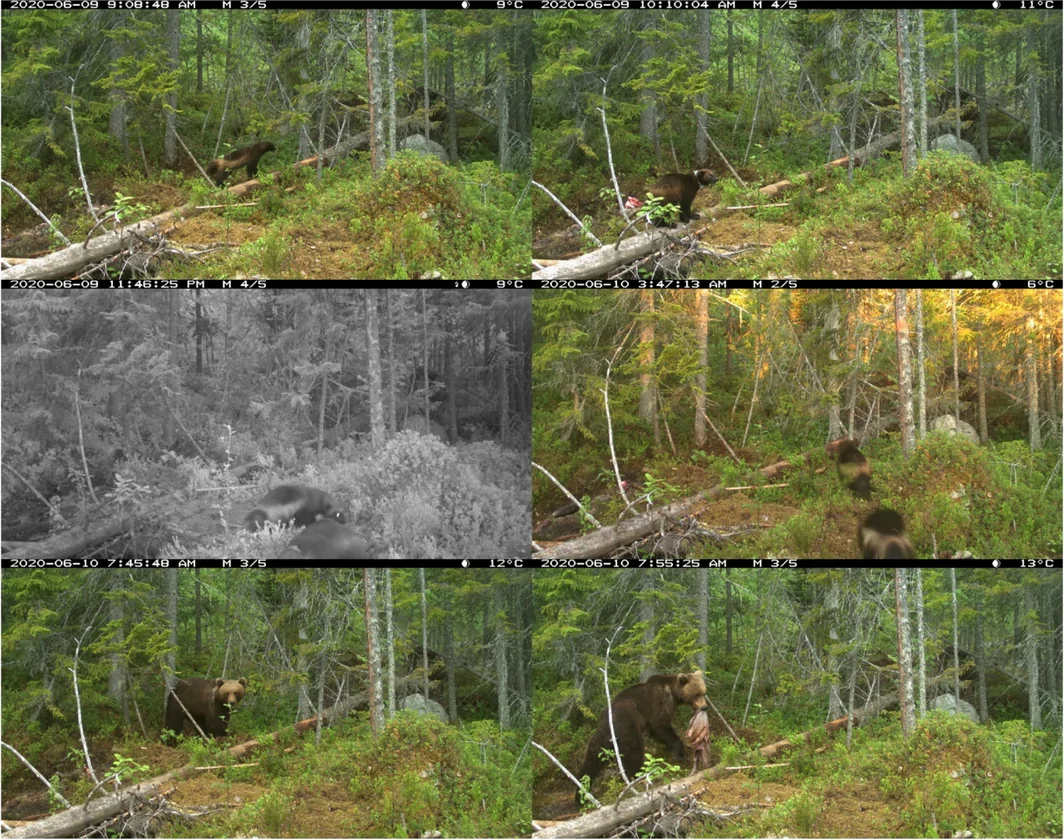
Photos from Site 4 (adult moose carcass). The top left photo shows an uncollared wolverine visiting the site. The top‐right photo shows the GPS‐collared male wolverine visiting the site. The middle‐left photo shows the GPS‐collared male (visible in the lower part of the photo) and an uncollared individual. The middle‐right photo shows two uncollared individuals, potentially mother and offspring. The lower‐left photo shows the bear arriving at the site, and the lower‐right photo shows the bear carrying away the carcass remains from the site.

The carcass was visited once by a bear (31 min; Figure 5). During this visit the bear moved the carcass, spent time feeding, and then left the site with most of the carcass remains. The bear arrived 2.5 h after a preceding wolverine visit at the site. One of the uncollared wolverines arrived at the site 34 min after the bear had left and started feeding on the leftovers of the carcass. When the camera was removed after 3 days (10/06/2020; Table 1), almost no carcass remains were left. However, based on the GPS‐data the collared male wolverine remained in the area for two more days and came back for a short visit 3 days later (Figure 1).

## 4 Discussion

Our observational study confirmed the occurrence of wolverine‐bear interactions during spring in central Sweden, an area with a high bear density and recently recolonized by wolverines. Wolverines regularly visited and spent significant time at or near bear‐killed moose carcasses, suggesting that wolverines were scavenging these bear‐kills. We documented bear activity at wolverine feeding sites and even at rendezvous sites used by wolverine females with cubs. However, we recorded no direct interactions between wolverines and bears.

The documented resource‐related interactions between both species in spring‐early summer need to be investigated in other seasons to fully understand the interspecific dynamics. Bears rely on a variety of food items, which change seasonally, but kill moose or scavenge carcasses not only in spring (Stenset et al. 2016). We found five adult moose presumably killed by bears, and wolverines spent much time at or near the two fresh carcasses (up to about 7 consecutive days < 3 km). This shows that wolverines are attracted to and use bear‐kills, which thus are a resource for wolverines and a cause for interspecific interactions between wolverines and bears. As food resources are especially crucial for female wolverines when reproducing and rearing cubs (Persson 2005; Inman et al. 2012), bear‐killed ungulate carcasses may provide valuable scavenging options, contributing to cub survival. Thus, similar to wolves providing moose carcasses further south in Scandinavia (van Dijk, Gustavsen, et al. 2008), and lynx providing reindeer carcasses in the north (Mattisson et al. 2016), bears might be an additional carrion provider for wolverines where they overlap, especially during spring.

All three monitored female wolverines had cubs and revisited the sites we determined as rendezvous sites. All three rendezvous sites monitored with camera traps were revisited by the female with cubs at least once. Additionally, at all rendezvous sites we found cached food remains, which were either eaten or retrieved by the female within a maximum of 7 days after camera placement. Using rendezvous sites, especially when caching food at the same location, might lead to elevated risk for both the female and offspring to encounter larger predators that are attracted to the cached food. This was illustrated by the fact that two rendezvous sites were visited by bears shortly before or after female wolverines with offspring used the site. Especially when cubs are left alone this may increase the risk that cubs are killed by bears, however, the cubs spent most of the time protected under rocks during this period. Also, we found that wolverine females bring cubs to carcasses that were presumably bear‐killed. If bear predation would be a significant risk for cubs, bringing them to a carcass to feed would be counterintuitive.

Wolverines are territorial species, and individual's territories usually only overlap with adult individuals of the opposite sex (i.e., intrasexual territoriality; Bischof et al. 2016; Hedmark et al. 2007). We observed two adult wolverines interacting at one of the moose carcasses, which were a male (GPS‐collared) and likely a female, considering the size difference and that the non‐aggressive behavior would likely not occur between unrelated individuals of the same sex. Furthermore, the uncollared female was accompanied by a cub on several occasions but never when the male was present at the site. Wolverine males are known to kill unrelated offspring (Persson et al. 2003), and mothers would likely avoid bringing cubs close to males they did not mate with. We did not observe direct interactions between the adult male, presumably the father, and the cub.

Several wolverine feeding sites contained remains from harvested moose (e.g., sawn‐off bones). Even though the moose hunting season in Sweden is in autumn (September–January; Wikenros et al. 2024), wolverines use hunting remains from slaughter piles (e.g., areas were remains are discarded) throughout the year (Gautier 2023). While we did not document the use by both bears and wolverines at the same sites, other studies found that bears commonly scavenge on hunting remains (Brown et al. 2023; Elfström et al. 2014), and we expect wolverine‐bear interactions to occur at these sites as well.

The wolverine recolonization into the boreal forest system (Persson et al. 2023) provides an opportunity to increase our understanding of mechanisms driving intra‐guild interactions and species coexistence. On the one hand, the occurrence of two or more predators and opportunistic scavengers may increase resource competition when using similar resources (Brogi et al. 2025; Moleón et al. 2014; Pereira et al. 2014; Tallian et al. 2022). We documented that bears and wolverines utilize similar food resources and that there is room for interactions between the two species at feeding sites, which highlights that bears can be a competitor for wolverines (Ordiz et al. 2020; Tallian et al. 2017, 2022). Bears, additionally, visited important sites for reproductive wolverine females and their offspring and, therefore, can pose a direct threat to wolverine cubs when attracted to wolverine cache sites. On the other hand, bear‐killed adult moose could provide locally and temporary important food resources for wolverines, especially during the cub‐rearing period. Thus, interactions may also involve facilitation (Mattisson, Andrén, et al. 2011). Further research should aim at disentangle the underlying mechanisms and potential effects of interspecific interactions in this boreal carnivore guild, which is one of the most complete in the world (Ripple et al. 2014).

## Author Contributions

**Rick W. Heeres:** conceptualization (equal), data curation (lead), investigation (equal), methodology (equal), writing – original draft (lead), writing – review and editing (equal). **Jens Persson:** conceptualization (equal), funding acquisition (equal), methodology (equal), project administration (equal), writing – review and editing (equal). **Johanna Heeres:** conceptualization (equal), investigation (equal), methodology (equal), writing – review and editing (equal). **Andrés Ordiz:** writing – review and editing (equal). **Jonas Kindberg:** writing – review and editing (equal). **Malin Aronsson:** conceptualization (equal), funding acquisition (equal), methodology (equal), project administration (equal), writing – original draft (equal), writing – review and editing (equal).

## Funding

FORMAS (Swedish Research Council for Sustainable Development, grant number 2016‐00924), Swedish Environmental Protection Agency (grant number 2020‐00110), Carl Tryggers Foundation (grant number CTS: 17:359), WWF Sweden (grant number 500475), and Marie‐Claire Cronstedt Foundation.

## Conflicts of Interest

The authors declare no conflicts of interest.

## Supporting information


**Figure S1:** Wolverine activity based on GPS data (blue dots and black lines) surrounding a recently (< 1 week) killed adult moose carcass in central Sweden.
**Table S1:** Overview of the results from the predation studies on five wolverines in central Sweden.
**Table S2:** Overview of data related to the deployed motion triggered cameras (*n* = 4) during the study period in 2020 in central Sweden.

**Appendix S1:** Visits cameras.

**Appendix S2:** Visits carcasses.

**Appendix S3:** ReadMe data.

## Data Availability

The data that supports the findings of this study are added as Supporting Information.
