# Supplementary material for: Resource‐Related Interactions Between Wolverines and Brown Bears in Scandinavia
Source: Ecol Evol. 2026 Oct 30;16(11):e74553. doi: 10.1002/ece3.74553 (PMC13722612; doi:10.1002/ece3.74553)
Supplement: Supplementary file 1 [file ECE3-16-e74553-s004.pdf]

Supplementary Materials

Appendix for: "Resource-related interactions between wolverines and brown bears in Scandinavia"

Rick W. Heeres, Jens Persson, Johanna Heeres, Andres Ordiz, Jonas Kindberg & Malin Aronsson

Corresponding authors: Malin Aronsson (malin.aronsson@slu.se).

Table S1: Overview of the results from the predation studies on five wolverines in central Sweden. Containing the sex, main area, number of visited clusters and found sites, year, and study period per individual.

**Wolverine**	**Sex**	**County**	**# cluster/sites**	**Intensive period**
J19026	M	Jämtland	43/63	28/05/2019 – 17/06/2019
J19027	F	Västernorrland	49/62	28/05/2019 – 17/06/2019
J16014*	F	Gävleborg	26/57	22/05/2020 – 11/06/2020
J20030	M	Gävleborg	38/52	22/05/2020 – 11/06/2020
J20032[a]*	F	Gävleborg	9/20	22/05/2020 – 30/05/2020
		Total	165/254	

[a] The collar of J20032 malfunctioned during the study period, which is therefore shorter compared to J16014 and J20030.
* females with offspring and for which potential rendezvous sites were investigated using motion triggered cameras (J20032 used site 1 and 2 and J16014 used site 3).

Table S2: Overview of data related to the deployed motion triggered cameras (*n*=4) during the study period in 2020 in central Sweden. Information is included on the site (number and type), placement duration, and the number of visits by wolverines and bears.

**Site**	**Site type**	**Placed**	**Retrieved**	**Days**	**# photos**	**# visits wolverine**	**# visits brown bear**
1	Rendezvous	27/05/2020	17/06/2020	22	385	5	2
2	Rendezvous	27/05/2020	17/06/2020	22	13 255	3	3
3	Rendezvous	08/06/2020	16/06/2020	9	197	3	0
4	Moose carcass	08/06/2020	10/06/2020	3	1285	18	1

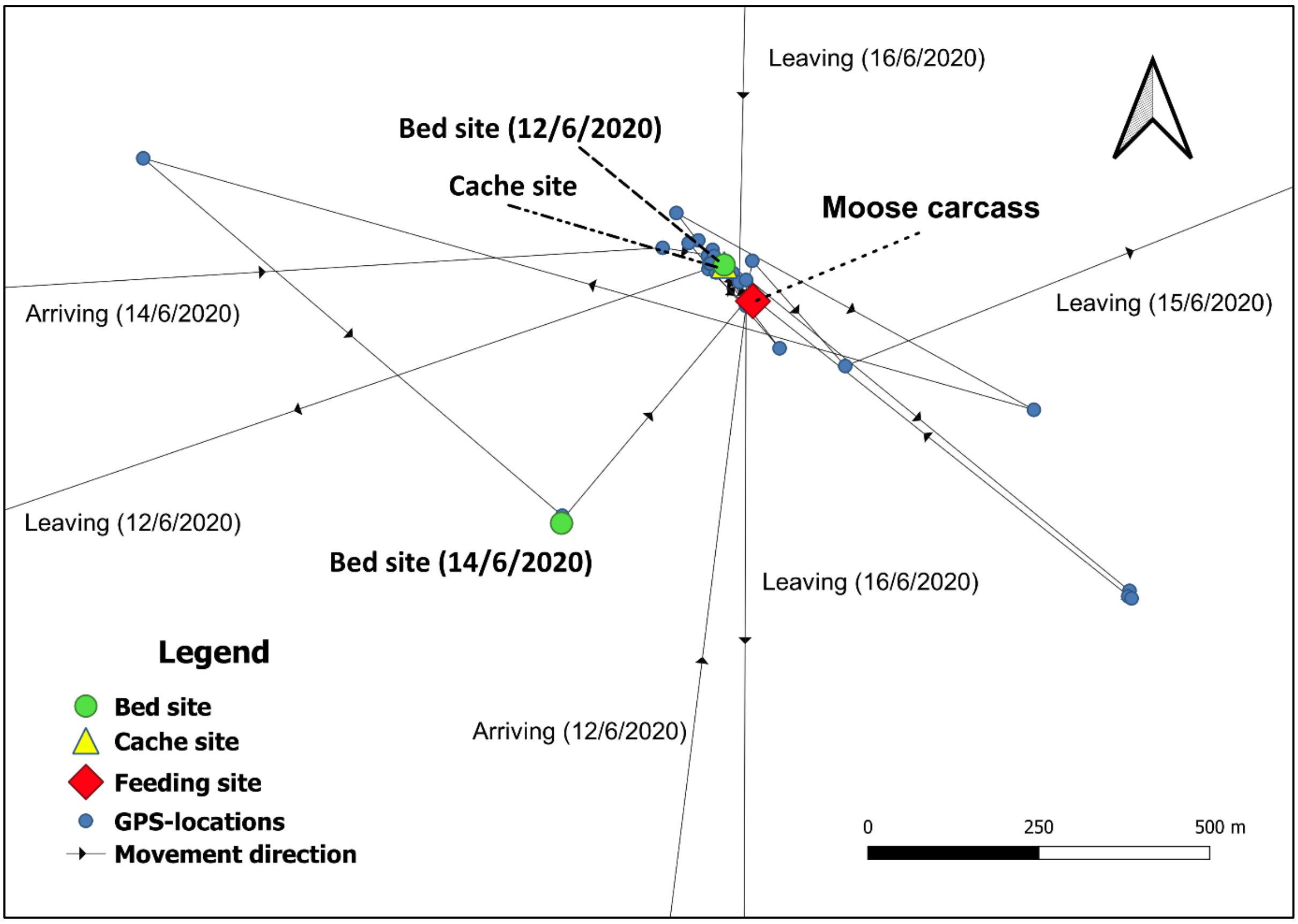

Figure S1: Wolverine activity based on GPS data (blue dots and black lines) surrounding a recently (< 1 week) killed adult moose carcass in central Sweden. Wolverine spent time at the feeding site (red square) 3 times: 2 times for almost 1 day (stays < 3 km from site on 12/6/2019 and 14/6/2019) and uses several bed and cache sites (green dots and yellow square, respectively), and 1 time (16/6/2019) for a couple of hours. Figure 2 (main document) shows another example of wolverine activity surrounding a recently killed moose carcass.
